# Effect of Non-Pharmacologic Vitamin D Status Correction on Circulating Bone Markers in Healthy Overweight and Obese Saudis 

**DOI:** 10.3390/molecules180910671

**Published:** 2013-09-02

**Authors:** Nasser M. Al-Daghri, Khalid M. Alkharfy, Abdulaziz Al-Othman, Sobhy M. Yakout, Yousef Al-Saleh, Mona Fouda, Shaun Sabico

**Affiliations:** 1 Biomarkers Research Program, Department of Biochemistry, College of Science, King Saud University, Riyadh 11451, Saudi Arabia; 2 Prince Mutaib Chair for Biomarkers of Osteoporosis, Biochemistry Department, King Saud University, Riyadh 11451, Saudi Arabia; 3 Department of Clinical Pharmacy, College of Pharmacy, King Saud University, Riyadh 11451, Saudi Arabia; 4 College of Applied Medical Sciences, King Saud University, Riyadh 11451, Saudi Arabia; 5 College of Medicine, King Saud University for Health Sciences, Riyadh 14611, Saudi Arabia; 6 Medicine Department, King Khalid University Hospital, Riyadh 12371, Saudi Arabia

**Keywords:** vitamin D, bone formation marker, osteocalcin, bone resorption marker, CTX, healthy adults

## Abstract

While moderate to severe vitamin D deficiency is prevalent in Saudi Arabia, skeletal effects associated with this deficiency are not common in this population. In this interventional study we measured the effects of improving vitamin D status on bone biochemical markers in overweight and obese adult Saudis. A total of 47 volunteers (21 males, 26 females) out of the initial 95 subjects were given verbal advice to expose themselves to sunlight for 5–30 min twice weekly and were encouraged to increase their intake of vitamin D–rich foods. Serum 25(OH)D, osteocalcin, and type 1 collagen cross-linked C-telopeptide (CTx), were measured at baseline and after one year. A significant decrease in the prevalence of vitamin D deficiency was observed (44% to 27%) after one year follow-up (*p* = 0.025). Also, a parallel significant increase in osteocalcin and a decrease in CTX and osteoprotegerin were observed. The results suggest that a modest increase in vitamin D levels among overweight and obese subjects through the promotion of lifestyle changes for one year have marginal effects in bone turnover markers as well as obesity itself.

## 1. Introduction

Bone turnover can be evaluated using a number of markers for bone formation and resorption [1]. Markers of osteoblastic bone formation include alkaline phosphatase and osteocalcin, whereas pro-collagen type 1 N-terminal peptide [PINP] is a marker of bone collagen formation [1], and collagen crosslinks (CTX-I, ICTP) are markers of bone resorption [1]. Markers of calcium metabolism, such as vitamin D metabolites and parathyroid hormone [PTH], are also important in assessing pathophysiological differences in the mechanisms of bone loss following physical inactivity [2]. Vitamin D stimulates intestinal calcium (Ca) and inorganic phosphorus (P) absorption, thereby protecting bone from resorption. 

Well-known consequences of vitamin D deficiency include secondary hyperparathyroidism, accelerated bone loss, increased bone turnover, proximal muscle weakness, and increase in body sway, falls, osteoporosis, and fractures [3]. Even in healthy populations of developed countries, hypovitaminosis D has been highly prevalent [4,5]. Many studies have shown that vitamin D levels in Saudi citizens are generally lower than the rest of the world, and one of the reasons is the equally high prevalence of overweight and obese Saudis [5,6]. Furthermore, although Saudi Arabia enjoys a sunny climate throughout the year, direct exposure to sun light of the local population is deficient due to climactic and cultural reasons. The limited sun exposure experienced by the residents of Saudi Arabia and the Gulf region as a whole can be compensated by vitamin D supplementation and increased dietary intake of vitamin D. While it is known that there is a low dietary intake of vitamin D globally secondary to a limited number of natural dietary sources, the benefits of increased vitamin D intake should be encouraged [7]. Adequate supply of vitamin D from skin synthesis and/or from dietary sources is considered essential for bone health [8,9].

Recently, Ardawi *et al*. demonstrated that vitamin D deficiency can affect bone turnover markers among Saudi Arabian men [10]. However, no further studies have been conducted as yet regarding the relationship between circulating bone biomarkers and vitamin D prospectively. In this study we aimed to investigate the correlations between these bone markers and vitamin D status over a period of one year. 

## 2. Results

**Figure 1:**
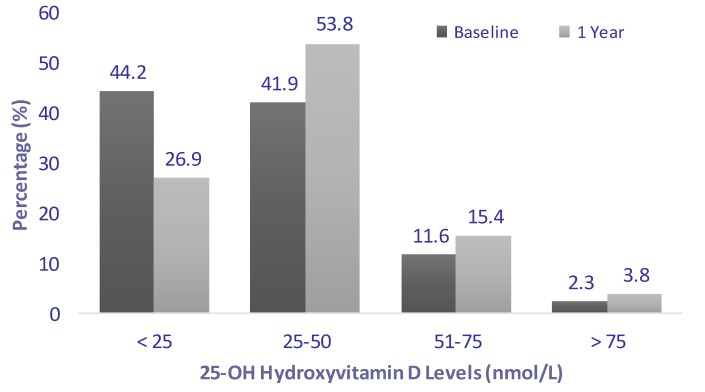
Decreasing prevalence of vitamin D deficiency over the 12-month intervention period.

Table 1 highlights the general characteristics of subjects. The most notable observations were the improved mean circulating levels of 25(OH)D and the parallel, significantly improved levels of calcium. Although the rest of the variables were not statistically significant, there was a modest but steady decrease of mean diastolic blood pressure and a decrease in low-density lipoprotein cholesterol levels over 12 months. Figure 1 reveals the significant decrease in the prevalence of vitamin D deficiency and the subsequent increase in the prevalence of vitamin D insufficiency and sufficiency (*p* = 0.025).

**Table 1:** General characteristics of subjects at baseline and 12 months post-intervention.

	Baseline	1 Year	P value
N	47	47	
Age (years)	44.9 ± 9.7		
Gender (M/F)	21/26		
BMI (kg/m^2^)	31.8 ± 3.3	32.4 ± 3.1	0.13
Systolic BP (mmHg)	116.5 ± 11.3	116.5 ± 17.2	0.94
Diastolic BP (mmHg)	79.5 ± 8.2	76.0 ± 9.9	0.06
Hip circumference (cm)	110.0 ± 9.2	112.8 ± 3.7	0.06
Waist circumference (cm)	101.0 ± 10.5	98.6 ± 9.6	0.25
Glucose (mmol/L)	5.4 ± 0.54	5.6 ± 0.60	0.09
Insulin (IU/mL)	19.0 ± 3.5	24.1 ± 5.2	0.16
Triglycerides (mmol/L)	1.4 ± 0.9	1.5 ± 0.84	0.57
Total Cholesterol (mmol/L)	4.9 ± 1.1	4.8 ± 0.85	0.62
HDL-Cholesterol (mmol/L)	0.91 ± 0.23	1.1 ± 0.39	0.005
LDL-Cholesterol (mmol/L)	3.5 ± 0.90	3.4 ± 0.80	0.57
Vitamin D (nmol/L)	30.3 ± 1.7	37.4 ± 1.5	0.001
PTH (pmol/L)	0.78 ± 0.12	0.77 ± 0.14	0.71
Albumin (g/L)	43.4 ± 4.6	41.7 ± 4.7	0.07
Calcium (mmol/L)	2.4 ± 0.15	2.5 ± 0.32	0.04
Corrected calcium (mmol/L)	2.7 ± 0.49	2.9 ± 0.56	0.06
Pi (mmol/L)	1.0 ± 0.29	1.1 ± 0.16	0.04
Crosslaps(ng/mL)	0.33 ± 0.16	0.23 ± 0.10	<0.001
Osteocalcin (ng/mL)	4.0 ± 1.2	5.1 ± 1.3	<0.001
Osteopontin (ng/mL)	9.2 ± 1.4	10.7 ± 1.1	0.23
Osteoprotegerin (pg/mL)	404.4 ± 16.5	354.8 ± 12.5	0.05

Note: P-value significant at *p* < 0.05.

Table 2 reveals the association of osteocalcin and crosslaps among the other variables measured over time. At baseline, significant associations of osteocalcin included calcium (R = −0.40), vitamin D (R = −0.45), and crosslaps (R = 0.42). One year after, osteocalcin levels were observed to be inversely and significantly associated with BMI (R = −0.59); diastolic blood pressure (R = −0.43), waist circumference (R = −0.44), calcium (R = −0.36) and corrected calcium (R = −0.43). Baseline associations of crosslaps on the other hand included total cholesterol (R = −0.51), and osteocalcin (R = 0.42). Other associations of crosslaps included inverse associations with hip circumference (R = −0.54) and PTH (R = −0.51). Positive associations were also noted between crosslaps and diastolic blood pressure (R = 0.44) and triglycerides (R = 0.40). The rest of the associations were unremarkable (Table 2).

**Table 2:** Associations of Selected Parameters using Osteocalcin and Crosslaps as Dependent Variables.

**Parameters**	**Osteocalcin**		**Crosslaps**	
	Baseline	1 Year	Baseline	1 Year
N	47		47	
BMI (kg/m^2^)	0.27	−0.59 *	−0.28	−0.36
Systolic BP (mmHg)	−0.005	−0.29	0.30	0.32
Diastolic BP (mmHg)	0.20	−0.43 *	0.12	0.44 *
Hips (cm)	0.21	−0.12	−0.07	−0.54 *
Waist (cm)	0.12	−0.44 *	0.13	−0.11
Insulin (IU/mL)	0.04	−0.32	0.17	−0.12
Calcium (mmol/L)	−0.40 *	−0.36	−0.36	−0.12
Corrected calcium (mmol/L)	−0.19	−0.43 *	0.32	0.16
Vitamin D (nmol/L)	−0.45 **	−0.32	0.21	0.18
PTH (pmol/L)	−0.01	0.13	−0.04	−0.51 *
Albumin (g/L)	0.07	0.24	0.01	0.06
Glucose (mmol/L)	0.003	−0.03	0.02	0.11
Triglycerides (mmol/L)	0.14	−0.06	−0.12	0.40 *
Total Cholesterol (mmol/L)	−0.06	−0.02	−0.51 **	0.10
HDL-Cholesterol (mmol/L)	−0.25	0.05	−0.15	−0.37
Pi (mmol/L)	−0.20	0.02	−0.39	−0.14
Osteocalcin (ng/mL)			0.42 *	−0.05
Osteopontin (ng/mL)	−0.20	−0.09	0.26	0.15
Crosslaps (ng/mL)	0.42 *	−0.05		
Osteoprotegerin (pg/mL)	0.27	0.08	−0.16	0.29

Note: Data is presented as coefficient R; * denotes significance at *p* < 0.05; ** denotes significance at *p* < 0.01.

## 3. Discussion

It has been assumed that populations living in sunny countries such as Saudi Arabia would be less likely to be vitamin D deficient due to abundant sunshine throughout the year. However, the results of this study, along with the earlier ones challenge such assumption. Vitamin D deficiency and insufficiency are very common in Saudis [25(OH)D ≤ 50 nmol/L]. 

Circulating bone biomarkers are useful for physicians to determine the bone quality of the patients. Many researchers have studied the correlation between these markers of turnover and bone mineral density (BMD) [11,12,13]. To date, there has been no conclusive evidence that an improvement in vitamin D status brings about a measurable change in overall bone turnover, an increase in bone formation or decrease in bone resorption as indicated by changes in bone markers. 

The present study found a strong correlation between 25-OH vitamin D and osteocalcin but not CTx at baseline. These markers are used to indicate bone turnover, and the correlations suggest that vitamin D stimulates osteoblasts to produce osteocalcin [14]. In the present study, there was a lack of PTH response to the intervention administered in this study. Secretion of PTH is regulated primarily by a drop in serum calcium [15,16] and does not appear to be affected by vitamin D levels until serum 25(OH)D concentrations fall below 30 ng/mL [16,17]. While this was not apparent in the study, several recent observations utilizing the same cohort found the same non-response of PTH despite alarmingly low vitamin D levels [10,18].

Osteoprotegerin and osteopontin are considered as bone metabolism biomarkers. Osteoprotegerin is the main osteoclastogenesis inhibitory modulator. It is also a member of tumor necrosis factor receptor (TNFR) super family produced by a variety of tissues. Osteoprotegerin acts as a soluble decoy receptor for a receptor activator of nuclear factor κ-B ligand (RANKL) and neutralizes this essential cytokine required for osteoclast differentiation [19]. Significant decreases of osteoprotegerin levels with increasing vitamin D in our study leads to inhibition of osteoclast differentiation and activity through blocking the interaction of RANK with its ligand (RANKL). The most prominent source of osteopontin is osteoblast. The expression of osteopontin and other non-collagenous proteins, such as osteocalcin, is highly regulated throughout osteoblast differentiation. In the present study, a modest increase was observed in circulating levels of osteopontin and a borderline significance was observed in osteoprotegerin after intervention. Furthermore, significant associations were elicited between osteocalcin and crosslaps with anthropometric indices such as BMI, waist and hip circumferences one year after intervention. These changes suggest that modest increase in vitamin D levels translate to alterations in bone remodeling among overweight and obese people, and further strengthens the hypothesis of a complex cytokine and hormonal crosstalk among bone cells, adipose tissue including the liver which affects both glucose, energy and bone metabolism [20]. The significant associations elicited between bone biomarkers, blood pressure and lipids aside from BMI post intervention also confirm the link between components of the metabolic syndrome, a cluster of cardiovascular risk factors, to bone metabolism. A recent meta-analysis highlight a strong association between metabolic syndrome and bone mineral density, at least in men, suggesting that metabolic syndrome is a risk factor for osteoporosis [21]. 

It is important to discuss how obesity itself, aside from vitamin D deficiency, contributes to altered bone biomarkers in the study population. It has been well established that lower vitamin D status observed among obese and overweight individuals is secondary to decreased bioavailability of vitamin D because of fat sequestration [22]. With respect to bone markers, inverse associations have been reported with regards to osteocalcin and and abdominal obesity in both men [23], and women [24], but the crosstalk between body fat mass and bone mass in general still needs further investigation.

The authors acknowledge several limitations one of which is the relatively small sample size which makes the findings at best, suggestive, and cannot be generalized. Other factors related to vitamin D status such as skin color and level of skin exposure to the skin were not taken into consideration. While all subjects were regularly monitored for sun exposure compliance, mean time sun exposure was not calculated as well as diet, and as such we cannot ascertain whether differences exist in the sun exposure time and intake between sexes.

In summary, the observed changes in bone turnover markers maybe secondary to the modest increase in vitamin D levels, but could be due to several unmeasured and uncontrolled factors. The intervention itself may have led to a significant decrease in vitamin D deficiency prevalence, but not enough to conclude that the changes in bone biomarkers has been solely because of vitamin D status correction. 

## 4. Experimental

### 4.1. Patients and Methods

This interventional study initially selected 95 subjects aged 30–60 years old to be included. Subjects are part of a larger cohort for vitamin D studies from the Prince Mutaib Chair for Biomarkers of Osteoporosis Research, King Saud University, Riyadh, KSA. For the purpose of this study, only those who were able to comply with the one year intervention were included. A total of 47 healthy volunteers (males = 21, females = 26) were able to complete the study, and the remaining 48 were excluded due to several reasons (lost to follow up, poor compliance, *etc*.). Volunteers have not taken any kind of medicine affecting bone metabolism for the last one month before the blood draw. Written informed consents were obtained before inclusion in the study. Ethics approval was granted by the Ethics Committee of the College of Science, King Saud University, Riyadh, Kingdom of Saudi Arabia (KSA). Participating subjects were recruited and enrolled longitudinally in four primary health care centers (PHCCs) within the Riyadh Central Region during the summer months (April–July 2009). They were asked to complete a generalized questionnaire, which contains demographic information, including past and present medical history, and to return after fasting for more than 10 h for anthropometry and blood withdrawal. They were also seen 12 months later for repeat assessments. At the screening visit, blood samples were examined for glycemic and lipid profile. Subjects who had abnormal levels of the variables measured were excluded. 

### 4.2. Anthropometry and Blood Collection

Subjects were requested to visit their respective PHCCs in an overnight fasted state (>10 h) for anthropometry and blood withdrawal by the PHCC nurse and physician on duty, respectively. Anthropometry included height (rounded off to the nearest 0.5 cm), weight (rounded off to the nearest 0.1 kg), waist and hip circumference (centimeters), and mean systolic and diastolic blood pressure (mmHg) (average of two readings). Body mass index was calculated as weight in kilograms divided by height in square meters. Fasting blood samples were collected and transferred immediately to non-heparinized tubes for centrifugation. Collected serum was then transferred to pre-labeled plain tubes; stored in ice; and delivered to the Biomarkers Research Program (BRP) in King Saud University, Riyadh, KSA, for immediate storage at −20 °C.

### 4.3. Sunlight Exposure and Vitamin D Diet

Subjects were given verbal advice to expose themselves to sunlight for 5 to 30 min twice a week either before 10:00 AM and/or after 3:00 PM (minimum body parts exposed were face, neck, hands and arms). The time for sun exposure was based on a previous study done by Hannan and colleagues [25] in Riyadh, KSA, detailing the hours of daylight during which ultraviolet radiation levels are considered carcinogenic and thus should be avoided. They were also regularly encouraged every week through Short Message Service (SMS) to take increased amounts of vitamin D–rich foods, such salmon, tuna, cow liver, dairy products, and vitamin D–fortified foods. To ensure compliance, they were instructed to keep a diary in which they recorded sun exposure times and outdoor physical activity; such diaries were submitted to the investigators at the end of the study period. Lifestyle modifications such as improved diet and increased physical activity were encouraged.

### 4.4. Sample Analyses

Fasting glucose, lipid profile, calcium, and phosphorous were measured using a chemical analyzer (Konelab, Espoo, Finland). Intact PTH and serum 25(OH)D were measured by a specific enzyme-linked immunosorbent assay (IDS, Tyne and Wear, UK). The inter- and intra-assay variabilities were 5.8% and 3.4% respectively for the intact PTH ELISA, 5.3% and 4.6% respectively for the 25(OH)D ELISA. Caution was exercised in the interpretation of results, as significant variability between different assays and laboratories has been reported [26]. It is noted that the BRP laboratory is an accredited laboratory by the Vitamin D External Quality Assessment Scheme (DEQAS). 

Serum C-terminal cross-linked telopeptide of type I collagen (CrossLaps, also known as b-CTx) and ostacalcin were estimated by Roche Elecsys modular analytics Cobas e411 using electrochemiluminescence immune-assays (Roche Diagnostics GmbH, Mannheim, Germany) using commercially available kits.

Other bone markers such as osteoprotegerin and osteopontin were measured using Luminex IS 200 (Lincoplex, Billerica, MA, USA) and performed as per the manufacturer’s instructions (Millipore, Billerica, MA, USA) with a detection across a range of 1–10,000 pg/mL for each analyte.

### 4.5. Data Analysis

Data were analyzed using the Statistical Package for the Social Sciences version 16.0 (SPSS, Chicago, IL, USA). Normal continuous variables were presented as mean ± standard deviation. Paired Student T-test was performed to compare differences between baseline and 1 year. Spearman correlation was done using osteocalcin and crosslaps as dependent variables. The *p*-values were adjusted for multiple testing using the Bejamini and Hochberg false discovery rate (FDR) method, which uses ranked p-values to determine the cut-off, at which point the type 1 error rate is below 0.05 [27]. Parameters with an FDR (q-value) > 0.05 were non-significant. 

## 5. Conclusions

We conclude that a modest increase in serum vitamin D levels among vitamin D deficient obese and overweight Saudis secondary to a one-year proposed changes in lifestyle seem to have a marginal role in affecting bone metabolism. It is possible that increased vitamin D supplementation, coupled with the promotion of outdoor physical activity which may help reduce BMI and further increase vitamin D levels to a corrected status are necessary to elicit the desired effect in bone turnover markers. 
